# Associated risk factors of underweight among under-five children in Ethiopia using multilevel ordinal logistic regression model

**DOI:** 10.4314/ahs.v21i1.46

**Published:** 2021-03

**Authors:** Nigussie Adam Birhan, Denekew Bitew Belay

**Affiliations:** 1 Department of Statistics, Mekdela Amba University, Tulu Awuliya, Ethiopia; 2 Department of Statistics, Bahir Dar University, Bahir Dar, Ethiopia

**Keywords:** Underweight, Partial proportional odds model, Multilevel partial proportional odds model, under-five children

## Abstract

**Background:**

Malnutrition is associated with both under nutrition and over nutrition which causes the body to get improper amount of nutrients to maintain tissues and organ function. Under nutrition is the result of insufficient intake of food, poor utilization of nutrients due to illnesses, or a combination of these factors. The purpose of this study was to identify associated risk factors and assess the variation of underweight among under-five children of different regions in Ethiopia.

**Methods:**

Ethiopian Demography and Health Survey (EDHS-2016) weight-to-age data for under-five children is used. In order to achieve the objective of this study; descriptive, single level and multilevel ordinal logistic regression analysis were used.

**Results:**

From a total of 8935 children about 8.1% were severely underweight, 17.1% were moderately underweight and 74.8% were normal. The test of heterogeneity suggested that underweight varies among region and multilevel ordinal model fit data better than single level ordinal model.

**Conclusion:**

Educational level of mother, religion, birth order, type of birth, sex of child, mother body mass index, birth size of child, existence of diarrhea for last two weeks before survey, existence of fever for last two weeks before survey, duration of breast feeding, age child and wealth index had significant effect on underweight among under-five children in Ethiopia. The finding revealed that among the fitted multilevel partial proportional odds model, the random intercept model with fixed coefficients is appropriate to assess the risk factors of underweight among under-five children in Ethiopia. The findings of this study have important policy implications. The government should work closely with both the private sector and civil society to teach women to have sufficient knowledge, awareness and mechanisms of improving under-five underweight for children's wellbeing.

## Background of study

Nutrition is a process by which individual achieve their physical and mental growth throughout their life cycle^1^. Adequate nutrition is important in early childhood to ensure healthy growth, learn new skills, neurological and mental development, think critically, contribute to their communities, and give children a better start in their life^2^.

Malnutrition is associated with both under nutrition and over nutrition which causes the body to get improper amount of nutrients to maintain tissues and organ function 3, 4. Malnutrition is the main health problem in developing countries and it account nearly 45% of children death and 11% loss of GDP every year^5^. Under nutrition is the result of insufficient intake of food, poor utilization of nutrients due to illnesses, or a combination of these factors. It is occurred due to significant deficiencies in any or all form of the energy, protein, or essential vitamins, minerals caused by a range of factors^6^, ^7^. It costs the economy of the world about 2 to 3 percent of GDP in every year^8^. The prevalence of underweight among under five years children were used to measure the burden of malnutrition 9.

Under nutrition remains a serious problem in many developing countries. It affects over 815 million children and cause of death for more than half of children. Around 195 million under-five children were affects by malnutrition; among those 90% were live in sub Saharan Africa and South Asia. Ethiopia is one of these countries with the highest prevalence of under-five mortality. At least 53% of children death can be caused directly or indirectly by malnutrition^10^.

In last the decades, Ethiopia planned to reduce child under nutrition, but significant improvement cannot obtain still due to multidimensional and complex factors. Yearly, under nutrition is causes the mortality of 24% of the children^11^.

A number of studies have been conducted to identify risk factors related to underweight among under five children in Ethiopia by using binary logistic regression^12^–^14^ and multilevel binary logistic regression^15^,^16^. However, binary logistic regression classifies underweight in to two categories to fulfill requirements of binary logistic regression. In this study the authors consider ordered classification of under nutrition to see the severity of children nutritional status in Ethiopia. Therefore, the objective of this study is to identify associated risk factors and assess the variation of underweight among under-five children of different regions in Ethiopia using multilevel ordinal logistic regression model.

## Methods

### Source of data

The source of data for this study was Ethiopian Demographic and Health Survey (EDHS 2016) which was obtained from Central Statistical Agency (CSA) under the auspices of the Ministry of Health. The survey was conducted from January 18, 2016 to June 27, 2016 based on a nationally representative sample that provides estimates at the national and regional levels.

EDHS sample was selected using a stratified, two-stage cluster design and enumeration areas were the sampling units for the first stage.

### Response variable

The response variable of the study was underweight which was measured by weight for age Z-score. Weight and age measurements of children were converted into Z-scores based on the National Center for Health Statistics (NCHS) reference population recommended by the World Health Organization (WHO). Underweight can be categorized into three groups: Severely underweight (Z-score< -3.00), moderately underweight (and normal (Z-Score -2.00). From the anthropometrics measures, Authors use weight-for-age to analysis underweight of children's. Weight-for-age is a composite index of height-for-age and weight-for-height and it is overall indicator of population's nutritional health. It takes into account both chronic and acute malnutrition and has a high positive predictive value as indicator of child malnutrition^17^, ^18^.

### Risk factors

The risk factors included in this study are grouped in to socio-economic, demographic, health, environmental and community factors.

**Socio-economic risk factors are:** - mother educational level, occupational status of mother, household wealth index, and duration of breast feeding status

**Demographic risk factors are:**- age of child, sex of child, marital status of mother, number of household member, age of mother at first birth, type of birth, Birth order, birth size and religion of mother.

**Environmental and community risk factors are:** - region, place of residence, toilet facility for household, and source of drinking water for household.

**Health related risk factors are:** - mother body mass index, existence of diarrhea in the two weeks before survey, existence of fever in the two weeks before survey, and existence of cough in the two weeks before survey.

### Statistical models

To analyze underweight among under-five children descriptive, cross tabulation, single level and multilevel ordinal logistic regression model techniques have been applied. In this study chi-square was applied to investigate the association between the underweight and each risk factor. Single level ordinal logistic regression models were conducted to identify the significant risk factors of underweight among under-five children in Ethiopia. Those models were proportional odds model, generalized ordered logit model, partial proportional odds model, continuation ratio model, adjacent-categories model and stereo type model^19^–^21^. Then other advanced models were applied.

### Testing of parallel lines

Ordinal logistic regression assumes that the coefficients that describe the relationship between the lowest versus all higher categories of the response variable are the same as those that describe the relationship between the next lowest category and all higher categories. This is called the proportional odds assumption. The test of parallelism contains -2log - likelihood for the constrained model, the model that assumes the planes or surfaces are parallel and -2log - likelihood for the general model, the model that assumes planes or surfaces are separated. The chi-square statistic is the log-likelihood difference between the two models. If the lines or planes are parallel, the observed significance level for the change should be large, the parallel model is adequate 22.

If the proportional odds assumption is not met, there are several options: the first is to collapse two or more levels, particularly if some of the levels have small sample size. The second is do bivariate logistic analyses, to see if there is one particular independent variable that is operating differently at different levels of the dependent variables. This can be done in various ways, including adjacent and global methods. The third is to use the partial proportional odds model. The last option is to use multinomial logistic regression 23.

In this study, single level ordinal logistic regression were extends to Multilevel Partial Proportional Odds Model (PPOM) to analyze the relationship between the underweight among under five years children and each of the independent variable which are included in the model and to compare variation with regard to underweight among and within geographical region of Ethiopia.

Multilevel model is used to allow not only independent variables at any level of hierarchical structure but also at least one random effect above level one group^24^. Multilevel ordinal logistic regression model is the analysis of hierarchical and ordinal dependent variable^25^. In this study, the clustering of the data points within geographical regions offers a natural 2-level hierarchical structure of the data, i.e. children are nested within regions. There are nine regions and two city administrations used in 2016 EDHS. Therefore, eleven geographical regions have been used for this study. The data were analyzed using STATA 14.0 and SPSS 21 software.

**Model I (Empty model):** This model is used as the baseline model for future model comparison and estimates the overall average of the outcome variables across all subjects and between-groups and with-in group variances. It was fitted without risk factors other than an intercept. According to 25 multilevel random intercept model for ordinal response variable using the logit link for the cumulative probabilities of being at or below a particular category of k for the ith child and jth region is given as follow:

$$\eta_{\mathrm{ij}}=\mathrm{logit}\text{(}\pi_{\mathrm{kij}})=\log\left(\frac{\pi (y_{\mathrm{ij}}\leq k)}{\pi (y_{\mathrm{ij}}>k)}\right)=\alpha_{k}-\beta_{\mathrm{oj}}$$

Where, β_0*j*_ = γ_00_ + μ_0*j*_

Result becomes

*η_ij_ = logit*(*π_kij_* (*y_ij_* ≤ *k*)) = *α_k_ − γ*_00_ − *μ*_0*j*_

Where, *γ*_00_ is the overall logit of being at or below a particular underweight across regions and it set zero since the cut off points *α_k_* estimated. Then the overall null model becomes

η_ij=logit(π_kij (y_ij≤k))=α_k-μ_0j

Where, µ_0j∼N(0,σ_μ0^2) and it is random term of regional level. Thus, *σ*^2^_*μ*0_ measures regional variations of under-five children underweight and also known as random intercept variance.

### Intra class correlation coefficient (ICC)

Intra class correlation coefficient is used as assessment of how much variations in the response categories lie at the level two (region level). The intra class correlation coefficient indicates the proportion of the variance explained by the grouping structure in the population. Its value is between 0 and 1. When the value is near to 0, multilevel model does not fit the data well. Whereas, if the value is large it shows that multilevel analysis required to handle this variation. When logistic model is used the residual at level one (child level) are assumed to follow the standard logistic distribution with mean 0 and variance $\frac{\pi^{2}}{3}=3.29$^25^, ^26^. It is expressed as:

$$\mathrm{ICC}=\frac{\sigma_{\mu 0}^{2}}{\sigma_{\mu 0}^{2}+\frac{\pi^{2}}{3}}$$

*σ*^2^_*μ*0_ is the variance of the higher level(regions).

Model II (Random Intercept Model): It is a model which includes level one risk factors in empty model. The intercept is allowed to vary randomly across regions and the slope for risk factors to be fixed to zero. In this study, multilevel partial proportional odds model is applied to relax the proportional odds assumption. Multilevel partial proportional odds model is the extension of partial proportional odds model which allows one or more independent variables have different effect on K-1 cumulative logits 27. The model is given as:

$$\eta_{\mathrm{ij}}=\mathrm{logit}\text{(}\pi_{\mathrm{kij}})=\log\left(\frac{\pi (y_{\mathrm{ij}}\leq k)}{\pi (y_{\mathrm{ij}}>k)}\right)=\alpha_{k}-\left(\beta_{0j}+\sum_{c=1}^{p}\beta_{\mathrm{cj}}x_{\mathrm{cij}}+\sum_{h=1}^{q}v_{\mathrm{kh}}w_{\mathrm{hij}}\right)$$

Hence,

*β*_0*j*_ = *γ*_00_ + *μ*_0*j*_, *β*_1*j*_ = *γ*_10_, *β*_2*j*_ = *γ*_20_, ..., *β*_*pj*_ = *γ*_*p*0_

Now the equation is written as:

$$\eta_{\mathrm{ij}}=\mathrm{logit}\left(\pi_{\mathrm{kij}}(y_{\mathrm{ij}}\leq k)\right)=\alpha_{k}-(\sum_{c=1}^{p}\gamma_{c0}x_{\mathrm{cij}}+\sum_{h=1}^{q}v_{\mathrm{kh}}w_{\mathrm{hij}}+\mu_{0j})$$

Where, $\alpha_{k}-(\sum_{c=1}^{p}\gamma_{c0}x_{\mathrm{cij}}+\sum_{h=1}^{q}v_{\mathrm{kh}}w_{\mathrm{hij}})$ is the fixed part of the model, μ_0j is the intercept variation of the model, *x*_1*ij*_,*x*_2*ij*_, ..., *x*_*pij*_ are the fixed risk factors which satisfies proportional odds assumption and γ_10,γ_20,…,γ_p0 are the coefficients of the fixed risk factors. w_hij is h x1 vector containing the value of observation ij of the set of h covariates for which proportional odds is not assumed and is *ν_kh_* hx1 vector of regression coefficient associated with *w_hij_* and it contains category k, due to this h covariates are allowed to vary across K-1.

### Random Coefficient Model

This is used to assess whether the slope of any of the explanatory variables has a significant variance component between the regions. This implies that the coefficients of explanatory variables are random at level two.

According to 27 multilevel partial proportional odds model for random coefficient model is given as:

$$\eta_{\mathrm{ij}}=\mathrm{logit}\left(\pi_{\mathrm{kij}}\right)=\log\left(\frac{\pi (y_{\mathrm{ij}}\leq k)}{\pi (y_{\mathrm{ij}}>k)}\right)=\alpha_{k}-(\beta_{0j}+\sum_{c=1}^{p}\beta_{\mathrm{cj}}x_{\mathrm{cij}}+\sum_{h=1}^{q}v_{\mathrm{kh}}w_{\mathrm{hij}})$$

Where,

*β*_0*j*_ = *γ*_00_ + *μ*_0*j*_, *β*_1*j*_ = *γ*_10_ + *μ*_1*j*_, *β*_2*j*_ = *γ*_20_ + *μ*_2*j*_, ..., *β*_*pj*_ = *γ*_*p*0_ + *μ_pj_*

Thus, the model becomes

$$\eta_{\mathrm{ij}}=\mathrm{logit}\left(\pi_{\mathrm{kij}}(y_{\mathrm{ij}}\leq k)\right)=\alpha_{k}-(\sum_{c=1}^{p}\gamma_{c0}x_{\mathrm{cij}}+\sum_{h=1}^{q}v_{\mathrm{kh}}w_{\mathrm{hij}}+\mu_{0j}+\sum_{c=1}^{p}\mu_{\mathrm{cj}}x_{\mathrm{cij}})$$

Where, $\alpha_{k}-(\sum_{c=1}^{p}\gamma_{c0}x_{cij}+\sum_{h=1}^{q}w_{hij}v_{kh})$ is the fixed part of the model, $\mu_{0j}+\sum_{c=1}^{p}\mu_{cj}x_{cij}$ is the random part of the model, α_k is the cut point of the model, *μ*_0*j*_ is intercept variation of the model, μ_1j,μ_2j,…,μ_pj slope variation of the model, γ_10,γ_20,…,γ_p0 are the coefficients of the risk factors. *w_hij_* is h x1 vector containing the value of observation ij of the set of h covariates for which proportional odds is not assumed and *ν_kh_* is hx1 vector of regression coefficient associated with *w_hij_* and it contains category k, due to this h covariates are allowed to vary across K-1.

## Results

### Descriptive analysis

The analysis was based on 8935 under-five children, among those 74.8%, 17.1% and 8.1% were normal, moderately underweight and severely underweight respectively in Ethiopia. From Table 1 underweight varies from region to region. The highest percentage of severely underweight is observed in Afar (17.02%) and moderately underweight in Benishangul Gumiz (22.37%). Whereas, the smallest percentage of severely underweight (0.25%) and moderately underweight (4.23%) is observed in Addis Ababa. Children living rural areas were moderately and severely underweight with (18.70%) and (9.09%) respectively. The prevalence of children who were born from non-educated mothers were severely (10.15%) and moderately (19.75%) underweight. Similarly, children who were born from poor families were severely (11.49%) and moderately (20.48%) underweight. The highest percentage of severely underweight was found for 4 and above birth order (9.74%) and the lowest percentage was found in first birth order (5.95%). The percentage of severely underweight for multiple births were (16.34%) and single birth (7.91%). The prevalence of severely underweight for male were (8.47%) and female (7.72%). Children who had been affected by diarrhea during the Last two weeks before the survey date were severely underweight (10.54%) and moderately underweight (18.67%).

**Table 1:** Results of descriptive summary for risk factors of underweight among under-five children in Ethiopia based on EDHS, 2016

Variable	Categories	Underweight				Person chi square(p-value)
	
		Normal (%)	Moderately underweight (%)	Severely underweight (%)	Total count	
Region	Tigray	76.67	18.45	4.88	943	298.05
	Afar	61.11	21.87	17.02	846	(<0.001)
	Amhara	71.14	20.69	8.17	894	
	Oromia	77.55	17.79	6.66	1381	
	Somali	73.25	17.61	9.15	1170	
	Benishangul	65.62	22.37	12.01	733	
	SNNPR	78.59	14.90	6.51	1121	
	Gambela	80.29	13.08	6.63	558	
	Harari	80.17	14.16	5.66	459	
	Addis Ababa	95.52	4.23	0.25	402	
	Dire Dawa	73.36	17.99	8.64	428	
Residence	Urban	86.17	10.16	3.67	1634	140.04
	Rural	72.21	18.70	9.09	7301	(<0.001)
Mother education	No education	70.10	19.75	10.15	5695	221.04
	Primary	80.27	14.38	5.35	2316	(<0.001)
	Secondary &higher	89.72	7.90	2.38	924	
Toilet	No	69.04	20.21	10.75	3999	134.22
	Yes	79.40	14.65	5.96	4936	(<0.001)
Religion	Orthodox	78.52	16.16	5.31	2710	75.62
	Muslim	71.39	18.32	10.28	4415	(<0.001)
	Other	77.35	15.69	6.96	1810	
Wealth index	Poor	68.03	20.48	11.49	4776	321.63
	Middle	75.62	18.09	6.29	1288	(<0.001)
	Rich	85.58	11.15	3.27	2871	
marital status	Married	74.75	17.14	8.10	8366	0.004
	Single	74.87	17.05	8.08	569	(0.998)
Mother occupation	No work	74.11	17.11	8.78	5249	7.98
	Had work	75.69	17.17	7.14	3686	(0.018)
Birth order	First	79.83	14.22	5.95	1765	63.68
	2–3	76.95	16.07	6.97	2868	(<0.001)
	4 & above	71.22	19.04	9.74	7302	
Birth type	Single	75.03	17.06	7.91	8733	22.09
	Multiple	63.37	20.30	16.34	202	(<0.001)
Sex child	Male	73.56	17.97	8.47	4568	7.22
	Female	76.02	16.26	7.72	4367	(0.027)
Birth size	Small	67.01	20.86	12.13	2498	142.53
	Average	75.88	16.99	7.14	3768	(<0.001)
	Large	80.44	13.86	5.70	2668	
Diarrhea	No	75.28	16.93	7.78	7901	12.64
	Yes	70.79	18.67	10.54	1034	(0.002)
Fever	No	75.28	16.71	8.01	7654	8.12
	Yes	71.66	19.67	8.67	1281	(0.017)
Cough	No	74.86	17.10	8.05	7445	0.27
	Yes	74.30	17.32	8.39	1490	(0.875)
Number of house hold member	4 & less	77.90	15.41	6.69	2421	21.34
	5–6	74.50	16.95	8.54	3079	(<0.001)
	7 and above	72.78	18.52	8.70	3435	
Body mass index	Thin	65.31	22.37	12.32	2110	205.95
	Normal	76.07	16.53	7.40	5983	(<0.001)
	Overweight	89.19	8.31	2.49	842	
Age child	Less than 6	88.27	7.07	4.66	1202	210.25
	6–11	83.27	10.85	5.88	765	(<0.001)
	12–23	74.32	16.75	8.93	1803	
	24–35	70.56	19.22	10.22	1722	
	36–47	70.87	20.57	8.55	1672	
	48–59	70.13	21.80	8.07	1771	
Mother age at 1^st^ birth	18 & less	72.79	18.60	8.61	4436	18.39
	>18	76.71	15.69	7.60	4499	(<0.001)
Duration of breast feeding	Ever breast	72.17	19.35	8.47	4614	42.33
	Never breast	72.84	18.81	8.36	335	(<0.001)
	Still breast	77.92	14.43	7.65	3986	
Source of water	Not improved	72.06	18.48	9.46	3561	25.55
	Improved	76.55	16.24	7.20	5374	(<0.001)

### Model Comparisons in Multilevel PPOM

The deviance value were used to select the best fitting model among the three fitted two-level partial proportional odds models. From Table 2, the deviance of the random intercept only model is 12854.148 and random intercept with fixed coefficient model is 11894.3346. This indicates that random intercept with fixed coefficient model is better than random intercept only model. And also, the deviance of random coefficient model is 11907.362. This also revealed that random intercept with fixed coefficient model is better than random coefficient model. Hence, random intercept with fixed coefficient model was a better fit as compared to random intercept only model and random coefficient model.

**Table 2:** Summary result for multilevel PPOM selection criteria

Tests	Random intercept only model	Random intercept with fixed coefficient model	Random coefficient model
Log likelihood	-6427.074	-5947.1673	-5953.681
Deviance	12854.148	11894.3346	11907.362
AIC	12860.148	11978.3346	11995.36
BIC	12881.44	12060.28057	12307.66

### Random Intercept Only Model

From Table 3 the empty model is considered as a parametric version of assessing heterogeneity of regions variance of the random effect (*σ*^2^_*μ*0_ = 0.596, S.E =0.345) and the Wald test statistic is (the square of the Z-ratio), W=(0.596/0.345)^2^ =2.98, which is compared with a chi-squared distribution on 1 degree of freedom is significant and *σ*^2^_*μ*0_ = 0.596 indicates that intercept variance across all regions. The hypothesis *H*_0_:*σ*^2^_*μ*0_ = 0 or there is no cross-regional variation for underweight among under-five children. For this hypothesis, the value of test statistic is 71.99 with p-value <0.001. Therefore, the null hypothesis is rejected and there is evidence of heterogeneity or cross regional variation of underweight among under-five children. This shows that an empty model for underweight among under-five children with random effect was better than an empty model for underweight among under-five children without random effect. Therefore, authors conclude that there was significant variation between regions for underweight among under-five children in Ethiopia.

**Table 3:** Result of parameter estimate of empty model

	Estimates	Std.Err	Z	p-value	95% Conf. Interval	
Cut1	1.020	0.034	30.42	0.000	0.955	1.086
cut2	2.377	0.045	52.71	0.000	2.289	2.465

Random-effects Parameters			Estimate	Std.Err	95% Conf. Interval	

Region:						
Var(_cons)			0.596	0.345	0.192	1.854

The intra class correlation coefficient (ICC) was obtained from intercept only model $\frac{0.596}{0.596+3.29}=0.153$ which is interpreted as 15.3% of variation in the underweight can be explained by grouping in regions (higher level units) and the remaining 84.7% of the variation is explained within region (lower level units).

LR test vs. ologit model: chibar2 (01) = 71.99 Prob >= chibar2 = 0.0000

From Table 3 the estimates of the fixed part of the model are 1.020 and 2.377 with p-value <0.001 which implies that the average log odds of underweight among under-five children are significantly different from zero. The average probability of under-five children being normal was $\frac{\mathrm{ex}p^{\text{1.020}}}{1+\mathrm{ex}p^{\text{1.020}}}=0.737$. Whereas, being normal or moderately underweight were $\frac{exp^{\text{2.377}}}{1+exp^{\text{2.377}}}=0.915$ which means the chance of being normal was 73.5% on average and chance of being normal or moderately underweight was 91.5 % on average.

### Random Intercept PPOM

The variance of random effect for random intercept multilevel model (0.093) is smaller than variance of random effect for empty random intercept model (0.596). The reduction of the random effect of the intercept variance is due to the inclusion of fixed risk factors. That is, the fixed independent variables can provide extra predictive value on underweight in each region.

In Table 4, multilevel PPOM revealed that mother education level, birth order, birth size, sex of child, birth type, body mass index of mother, duration of breast feeding status, age of children, house hold wealth index and religion of mother, existence of diarrhea and fever last week before survey date were significantly associated with underweight among under five children. From the significant variable age of child, house hold wealth index and religion of mother were failed the assumption of proportional odds model.

**Table 4:** Result of random intercept with fixed coefficients PPOM of underweight among under- five children in Ethiopia using the EDHS 2016

Risk factors that satisfied parallel line assumption							

Variables	Coeff.	Std.Err	Z	p-value	OR	95% CI OR	
Moedu(ref=no education)	0				1		
Primary	-0.227	0.068	-3.35	0.001	0.797*	0.698	0.910
Secondary and above	-0.497	0.133	-3.72	0.000	0.608*	0.468	0.790
Moccu(ref=had no work)	0				1		
Had work	-0.056	0.057	-0.99	0.322	0.945	0.845	1.057
Residence(ref=urban)	0				1		
Rural	-0.132	0.105	-1.26	0.208	0.877	0.714	1.076
Toilet(ref=no)	0				1		
Yes	-0.081	0.064	-1.26	0.207	0.922	0.814	1.046
Border(ref=first)	0				1		
2–3	0.074	0.082	0.90	0.368	1.077	0.916	1.265
4 and above	0.229	0.092	2.49	0.013	1.257*	1.050	1.505
Btype(ref=single birth)	0				1		
Multiple birth	0.700	0.157	4.45	0.000	2.013*	1.479	2.740
Sexchild(ref=male)	0				1		
Female	-0.165	0.051	-3.25	0.001	0.848*	0.767	0.936
Birthsize(ref=large)	0				1		
Average	0.276	0.065	4.28	0.000	1.318*	1.162	1.496
Small	0.142	0.069	9.29	0.000	1.900*	1.659	2.175
Diarrhea(ref=no)	0				1		
Yes	0.253	0.082	3.10	0.002	1.288*	1.097	1.511
Fever(ref=no)	0				1	
Yes	0.154	0.075	2.05	0.040	1.167*	1.007	1.352
BMI(ref=thin)	0				1		
Normal	-0.463	0.058	-8.02	0.000	0.630*	0.562	0.705
Over weight	-1.057	0.129	-8.18	0.000	0.347*	0.270	0.448

Water(ref=not improved)	0				1		
Improved	0.097	0.056	1.75	0.080	1.102	0.988	1.229
NoHH(ref=less than 5)	0				1		
5–6	-0.027	0.075	-0.36	0.716	0.973	0.840	1.127
Above 6	-0.053	0.084	-0.63	0.528	0.948	0.804	1.118
Durbreast(ref=ever breast)	0				1		
Never breast	0.105	0.133	0.79	0.429	1.111	0.856	1.441
Still breast	0.310	0.078	3.94	0.000	1.363*	1.169	1.590
Moth-age(ref=18 & less)	0				1		
Greater than 18	0.008	0.052	0.15	0.883	1.008	0.910	1.116

**Risk factors that does not satisfied parallel line assumption**							

**Normal versus (moderately underweight and severely underweight)**							

Agechild(ref=less than 6)	0				1		
6–11	0.415	0.137	3.04	0.002	1.514*	1.158	1.981
12–23	1.026	0.110	9.36	0.000	2.790*	2.251	3.459
24–35	1.381	0.118	11.68	0.000	3.979*	2.251	3.459
36–47	1.448	0.127	11.38	0.000	4.255*	3.317	5.463
48–59	1.550	0.130	11.91	0.000	4.711*	3.651	6.078
Windex(ref=poor)	0				1		
Middle	-0.251	0.082	-3.07	0.002	0.778*	0.663	0.913
Rich	-0.610	0.086	-7.06	0.000	0.543*	0.458	0.643
Religion(ref=orthodox)	0				1		
Muslim	0.285	0.093	3.08	0.002	1.330*	1.110	1.595
Other	0.278	0.108	2.58	0.010	1.320*	1.069	1.631
Constant	-3.289	0.485	-6.78	0.000	0.037*	0.014	0.097

**(Normal and moderately underweight) versus severely underweight**							

Agechild(ref=less than 6)	0				1		
6–11	0.255	0.208	1.23	0.220	1.290	0.858	1.941
12–-23	0.723	0.162	4.45	0.000	2.061*	1.498	2.832
24–35	1.009	0.167	6.05	0.000	2.743*	1.978	3.804
36–47	0.889	0.177	5.03	0.000	2.433*	1.719	3.442
48–59	0.925	0.179	5.16	0.000	2.522*	1.774	3.582
Windex(ref=poor)	0				1		
Middle	-0.437	0.130	-3.36	0.001	0.646*	0.501	0.834
Rich	-0.877	0.129	-6.79	0.000	0.416*	0.323	0.536
Religion(ref=orthodox)	0				1		
Muslim	0.593	0.124	4.78	0.000	1.809*	1.419	2.307
Other	0.494	0.148	3.33	0.001	1.639*	1.226	2.192
Constant	-0.150	0.686	-0.22	0.827	0.861	0.224	3.307

Random effect parameter		Estimate		Std.Err			

Level 2(region)							
Var (region):		0.093		0.050			

* Significant at 0.05, ref=indicates reference category

The odds of severely underweight for child from mother who had primary education were 0.797(OR=0.797, 95% CI: 0.698–0.910) and secondary and above education were 0.608 (OR= 0.608, 95% CI: 0.468–0.790) times lower than the odds of severely underweight for child from mother who had no education. The odds of having severely underweight for children with birth order 4 and above were 1.257 (OR=1.257, 95% CI: 1.050–1.505) times higher than the odds of severely underweight for children with first birth.

Children having a multiple birth was 2.013 times (OR=2.013, 95% CI: 1.479–2.740) more likely to severely underweight than those having single birth. Male children were 1.179 times (OR = 0.848, 95% CI: 0.767–0.936) more likely to be severely underweight than females. The odds of severely underweight for child born with average size were 1.318 (OR=1.318, 95 % CI: 1.162–1.496) and smaller than average size were 1.900 (OR=1.900, 95% CI: 1.659–2.175) times higher than the odds of severely underweight for child born with larger than average size. The odds of severely underweight for under-five children who had diarrhea in the last two weeks before date of survey were 1.288 (OR=1.288, 95 % CI: 1.079–1.511) times higher than the odds of severely underweight for children those who had no diarrhea in the last two weeks before date of survey. Children who had fever in the last two weeks before the survey time were 1.167 times (OR=1.167, 95 % CI: 1.007–1.352) more likely to be severely underweight than had no fever.

The odds of severely underweight for child from mother who had normal body mass index were 0.630 (OR=0.630, 95 % CI: 0.562–0.705) times and overweight body mass index were 0.347(OR=0.347, 95 % CI: 0.270–0.448) times the odds of severely underweight for child from mother who had thin body mass index. The odds of severely underweight for child who still breast feed were 1.363 (OR=1.363, CI: 1.169–1.590) times the odds of severely underweight for child who had ever breast feed.

When there is a variable which does not satisfied parallel line assumption the interpretation is in each cut points separately. The odds of moderately and severely underweight for child with age group 6–11, 12–23, 24–35, 36–47, and 48–59 were 1.514 (OR=1.514, 95% CI: 1.158–1.981), 2.790 (OR=2.790, 95% CI: 2.251–3.459), 3.979 (OR=3.979, 95% CI: 2.251–3.459), 4.255 (OR=4.255, 95% CI: 3.317–5.463), and 4.711 (OR=4.711, 95% CI:3.651–6.078) times the odds of moderately or severely underweight with age group less than 6 respectively. However, the odds of severely underweight for child with age group 12–23, 24–35, 36–47 and 48–59 were 2.061 (OR=2.061, 95 % CI: 1.498–2.832), 2.743 (OR=2.743, 95% CI:1.978–3.804), 2.433 (OR=2.433, 95% CI: 1.719–3.442), and 2.522 (OR=1.774, 95% CI: 1.774–3.582) times the odds of severely underweight for child age group less than 6 respectively. The odds of moderately or severely underweight for children who reside from family with middle wealth index were 0.778 (OR= 0.778, 95 % CI: 0.663–0.913) times and rich wealth index were 0.543 (OR= 0.543, 95% CI: 0.450, 0.643) times the odds of moderately or severely underweight for child born from poor wealth index family. The odds of severely underweight for child who resided in middle wealth index family were 0.646 (OR= 0.646, 95 % CI: 0.501–0.834) and rich wealth index family were 0.416 (OR= 0.416, 95 % CI: 0.323–0.536) times the odds of severely underweight for child who resided in poor family controlling for other variables in the model and random effect at level two.

## Discussion

This study attempted to identify determinants of underweight among under-five children in Ethiopia using EDHS 2016. Authors' findings reveal that among 8935 children, 8.1 % and 17.1 % were severely and moderately underweight respectively in Ethiopia. Authors used brant test and model selection criteria to select the better model among the fitted single level ordinal logistic regression model. Partial proportion odds model was the most appropriate and better model to analyzed underweight among under five children. With the help of chi-square test, covariates included in the study were significantly associated with underweight except existence of cough two weeks before survey and marital status at 25% significance. The significant variables were included in multivariable analysis. Multilevel partial proportional odds model technique was applied to analyze risk factors and variation of underweight within and among geographical regions of Ethiopia. The finding revealed that educational level of mother, religion, birth order, type of birth, sex of child, mother body mass index, birth size of child, existence of diarrhea for last two weeks before survey, existence of fever for last two weeks before survey, duration of breast feeding, age of the child and house-hold wealth index were significantly associated with underweight and there was significant variation between regions on underweight among under-five children in Ethiopia.

The finding of this study revealed that educational level of mothers had a significant effect with underweight. Children who were born from educated mothers are less risk on underweight in line with other studies 28, 29. Similarly, as birth order increases the risk of underweight also increase. This study is supported by a studies conducted by 30, 31. The current study found that the risk of underweight among under-five children were significantly associated with type of birth. Underweight of children having multiple births were high relative to single birth. This study is similar with the previous study^32^. This study revealed that female child has low risk of being underweight. This study is in lined with other studies 31, 33, 34. The result of this study also suggested that Small size children at birth were more severely underweight as compared to large size children. This finding is consistent with other study^35^. The finding of this study also showed that children who had fever two weeks before survey date were significantly vulnerable to underweight than those who had not. This finding is consistent with other studies^30^,^36^. Similarly, children who had diarrhea two weeks before survey date were more risk of underweight. This has been confirmed by different studies 13, 14. This study showed that women having high body mass index were less likely to be in higher levels of underweight among under-five children as opposed to lower levels of underweight. This result is consistent with the result of studies 30, 37. Children whose breast feeding status is still were more risks for underweight as compared to ever breast feeding. This finding is consistent with other studies^18^, ^38^.

In this study age of child was found to be significantly associated with underweight, as age of child increases the risk of being underweight increases. This finding seemed to be consistent with other studies 10,12,29,39. This study also revealed that the odds of severely underweight of children were relatively lower for children belonging to rich category of the wealth index as compared to poor. This finding is consistent with other studies 9, 15. The result of this study indicated that children born from other religions follower have higher chances of experiencing underweight compared to those Orthodox Christianity followers. This is consistent with the study 40.

## Conclusion

The significant risk factors for underweight among under five children analyzed using PPOM were residence, region, education level of mother, birth order, type of birth, sex of child, mother BMI, birth size of child, diarrhea for last two weeks before survey, fever for last two weeks before survey and duration of breast feeding by fulfilling brant test (proportional odds assumption). However, age of the child, house hold wealth index and religion failed the proportional odds assumption.

The covariates educational level of mother, religion, birth order, type of birth, sex of child, mother body mass index, birth size of child, existence of diarrhea for last two weeks before survey, existence of fever for last two weeks before survey, duration of breast feeding, age child and house hold wealth index had significant effect on underweight among under-five children in Ethiopia.

The findings of this study have important policy implications. The government should work closely with both the private sector and civil society to teach women to have sufficient knowledge, awareness and mechanisms of improving under-five underweight to make children very well. Design and implement primary health care and support the house holds to develop their economy.

## Abbreviations

CSA: Central Statistical Agency
EDHS: Ethiopian Demographic Health Survey
GDP: Gross Domestic Product
ICC: Intra-class Correlation Coefficient
NCHS: National Center for Health Statistics
PPOM: Partial Proportional Odds Model

## Declarations

### Availability of data and material

The datasets have been taken from Ethiopian Central Statistical Agency.
